# Leveraging the EHR4CR platform to support patient inclusion in academic studies: challenges and lessons learned

**DOI:** 10.1186/s12874-017-0299-3

**Published:** 2017-02-28

**Authors:** Yannick Girardeau, Justin Doods, Eric Zapletal, Gilles Chatellier, Christel Daniel, Anita Burgun, Martin Dugas, Bastien Rance

**Affiliations:** 1 Biomedical Informatics and Public Health department, Hôpital Européen Georges Pompidou, AP-HP, 10 Rue Leblanc, 75015 Paris, France; 2 Sorbonne Universités, UPMC Univ Paris 06, UMR_S 1138, Centre de Recherche des Cordeliers, F-75006 Paris, France; 3 Université Paris Descartes, Paris, France, Paris Sorbonne Cité, Paris, France; 4 Assistance Publique - Hôpitaux de Paris, Unité d’épidémiologie et de recherche clinique, Hôpital européen Georges Pompidou, Paris, France; 5 INSERM, U1142, LIMICS, AP-HP, F-75006 Paris, France; 6 Institute of Medical Informatics, University of Münster, Münster, Germany

**Keywords:** Clinical trial, Patient recruitment, Electronic health records, Clinical trial recruitment system

## Abstract

**Background:**

The development of Electronic Health Records (EHRs) in hospitals offers the ability to reuse data from patient care activities for clinical research. EHR4CR is a European public-private partnership aiming to develop a computerized platform that enables the re-use of data collected from EHRs over its network. However, the reproducibility of queries may depend on attributes of the local data. Our objective was 1/ to describe the different steps that were achieved in order to use the EHR4CR platform and 2/ to identify the specific issues that could impact the final performance of the platform.

**Methods:**

We selected three institutional studies covering various medical domains. The studies included a total of 67 inclusion and exclusion criteria and ran in two University Hospitals. We described the steps required to use the EHR4CR platform for a feasibility study. We also defined metrics to assess each of the steps (including criteria complexity, normalization quality, and data completeness of EHRs).

**Results:**

We identified 114 distinct medical concepts from a total of 67 eligibility criteria Among the 114 concepts: 23 (20.2%) corresponded to non-structured data (i.e. for which transformation is needed before analysis), 92 (81%) could be mapped to terminologies used in EHR4CR, and 86 (75%) could be mapped to local terminologies. We identified 51 computable criteria following the normalization process. The normalization was considered by experts to be satisfactory or higher for 64.2% (43/67) of the computable criteria. All of the computable criteria could be expressed using the EHR4CR platform.

**Conclusions:**

We identified a set of issues that could affect the future results of the platform: (a) the normalization of free-text criteria, (b) the translation into computer-friendly criteria and (c) issues related to the execution of the query to clinical data warehouses. We developed and evaluated metrics to better describe the platforms and their result. These metrics could be used for future reports of Clinical Trial Recruitment Support Systems assessment studies, and provide experts and readers with tools to insure the quality of constructed dataset.

**Electronic supplementary material:**

The online version of this article (doi:10.1186/s12874-017-0299-3) contains supplementary material, which is available to authorized users.

## Background

The research and development of new drugs requires the implementation of long, complex and costly processes [1]. Only clinical trials can provide the evidence of effectiveness and prove the safety of a new drug before commercialization. The design, deployment, and management of large scale trials come at a high cost. In 2010, the cost for the development of a new drug from early research to regulatory approval was estimated to be from $161 million to $1.8 billion. Approximately half of this amount was attributable to the clinical phase [1]. In this context, new approaches have been developed to reduce the cost of drug research and development studies [2].

The growing development and use of Electronic Health Records (EHRs) in hospitals offers the ability to reuse data from patient care activities for clinical research [3–5]. EHRs may include many types of data, including demographics data, medical history, medications, laboratory test results, radiology images, vital signs, and billing information. These data can be used to aid the three steps of a clinical trial: to identify eligible patients, to feed case report forms for ongoing studies, and to more effectively identify and notify adverse drug reactions [6]. However, EHR software and structure are neither dedicated nor adapted for clinical research.

Clinical Trial Recruitment Support Systems (CTRSS)s have been developed over the past decade to reuse data from EHRs for clinical research purpose [7]. CTRSSs can translate a set of eligibility criteria into a set of computable queries that can be run against an EHR to identify suitable patients for recruitment [8–11]. CTRSSs can be used to evaluate protocol feasibility (i.e. to identify and track patients with respect to inclusion criteria for future studies) and help conduct studies. CTRSSs provide access to patient data throughout the hospital (they are not limited to one service or one department) and may identify more eligible patients than traditional methods and increase the accrual rate of studies. Beauharnais has suggested that CTRSSs could decrease the cost of drug development by decreasing the time spent to deploy a study and improving clinical recruitment [12].

There are many CTRSSs with various levels of coverage [13]: locally for one research center, regionally (for a group of clinics), or internationally. The implementation of a given CTRSS at a specific site depends on many factors (for example the domain covered, or the standard terminology used). Comparing the usability of platforms can be difficult. Indeed, the deployment of such platforms raises new and complex issues. For example, how to translate eligibility criteria, usually expressed as free text, into computable formal queries that can be run against EHRs, or whether primary care data can be used to support research, and to what extent.

EHR4CR (Electronic Health Records for Clinical Research) is one of the largest public-private partnerships aiming to develop a computer platform to enable the reuse of data collected from EHRs located in university hospitals across Europe to support clinical research at all steps of the process. The objective of this project is to develop, deploy, and assess an innovative computer platform capable of simplifying all the tasks of CTRSSs.

The EHR4CR model has been developed and assessed on studies from industrial EFPIA (European Federation of Pharmaceutical Industries and Associations) partners [14]. While industry-sponsored studies mainly aim to assess the efficacy of new drugs, academic studies often cover a broader range of medical fields (e.g. non-interventional studies for routine care, new surgical procedures, or the assessment of new diagnostic tools). Such differences could affect the platform’s deployment and its final performance. Consequently, it was crucial to assess whether the EHR4CR platform could also be used in the specific context of institutional clinical studies.

Köpcke et al. performed a systematic literature review of CTRSS research published up to the end of 2013 [13]. They analyzed 101 papers on 79 different systems and concluded that “the success of a CTRSS depends more on its successful workflow integration than on sophisticated reasoning and data processing algorithms”. They highlighted the fact that most of the CTRSS reports suffered from the lack of a description of meaningful outcomes such as intermediary criteria representation, terminologies used, and the availability of patient data. They proposed a list of 15 items to ensure that future CTRSS design, implementation, and evaluation studies are “sufficiently described to allow researchers to better learn from other’s experience”.

Our objective was 1/ to describe and to provide outcomes on the different steps that were achieved using the EHR4CR platform for the support of our institutional studies, 2/ to identify the specific issues that could affect the final results of the platform.

## Methods

### HEGP and UKM clinical data warehouses

HEGP (Georges Pompidou European Hospital) is a 700 bed teaching hospital in Paris, France. HEGP deployed a clinical data warehouse (CDW) based on the i2b2 framework in 2008. The CDW collects from the hospital information system structured data (data directly reusable for further analysis, e.g. demographics, diagnosis codes, procedures, drug prescriptions, laboratory test results…) and unstructured data (i.e. data that need to be transformed before secondary use, including, but not limited to data stored in free-text format such as radiology reports or discharge summaries) [15, 16]. The CDW has been a support for the deployment of the EHR4CR platform at HEGP.

UKM (Munster University Hospital) is a 1400 bed teaching hospital in Münster, Germany. The Westfälische Wilhelms-Universität Münster (WWU) set up a CDW for the EHR4CR project with the project’s “native DB schema”. Because the CDW is not productively used, various tools have been developed to evaluate various tasks and scenarios of the project. There are currently “extract transform load” (ETL) scripts for subjects, diagnoses, procedures, tumor node metastasis classification, clinical parameters, and medications.

### Local terminologies (Table 1)

**Table 1:** The European Hospital Georges Pompidou (HEGP) and the University Hospital of Münster (UKM) Electronic Health Records Terminologies

Category	Terminology used	
	HEGP	UKM
Biological results	*Local (partially mapped to LOINC)*	*Local*
Clinical parameters (pulse, temperature, …)	*Local*	*Local*
Diagnosis (final discharge)	*ICD-10*	*ICD10-GM*
Medical Procedures	*CCAM (French medical procedure coding system)*	*OPS (German procedure coding system)*
Drug Prescriptions	*ATC*	*Local*
Clinical Reports	*None (free-text)*	*None (free-text)*
Demographic data	*Local*	*Local*
Complementary test reports	*None (free-text)*	*None (free-text)*
Pathological diagnosis	*ADICAP (French terminology for pathology)*	*TNM*
Chemotherapy Prescriptions	*Local*	*ATC*

Local terminologies used at HEGP and UKM for the project are described in Table 1.

### EHR4CR architecture

The EHR4CR platform is based on a distributed service-oriented architecture on which several components are connected to provide a collection of services for faster clinical studies management. The EHR4CR platform includes a central workbench where the final user can (i) write eligibility criteria by selecting standardized codes [17] (SNOMED-CT, ICD-10, ATC, LOINC, etc.), (ii) combine them with Boolean logic (AND, OR, NOT) and temporal (AFTER, BEFORE) operators, and (iii) store the script in a dedicated formal representation: the ECLECTIC (Eligibility Criteria Language for Clinical Trial Investigation and Construction) language [18]. For feasibility studies, the constructed query is submitted to a set of selected hospitals by the EHR4CR Orchestrator component. The query is received at the hospital by the EHR4CR endpoint. This component transforms the initial ECLECTIC query into a collection of SQL (Structured Query Language) queries run onto the hospital local CDW. Before the SQL queries are processed, the standardized codes may be converted into specific local codes by using the EHR4CR local mapping server. The patient numbers are sent back to the EHR4CR Central Workbench by the EHR4CR Orchestrator which aggregates the results to provide the number of patients matching each eligibility criterion in the different sites.

### Selected studies

We selected three institutional studies: two conducted at HEGP (aXa, DERENEDIAB) and one at UKM (EWING 2008):

- The aXa study [clinicaltrial.gov id: NCT02898051] is a prospective case–control study with a high enrollment rate. The goal of the aXa study is to determine whether the pharmacokinetics of low molecular weight heparin is predictive of recurrent thromboembolism in cancer subjects.
- The DERENEDIAB study [clinicaltrial.gov id: NCT01588795] is a multi-center, prospective, open, randomized, controlled study, with a low enrollment rate, to assess the effectiveness of renal denervation in addition to standardized medical treatment in diabetic subjects with severe diabetic nephropathy.
- The EWING 2008 study [clinicaltrial.gov id: NCT00987636] is an interventional, phase 3, multi-center, randomized, controlled, international study on Ewing Sarcoma.
  Number of criteria are described in Table 2 for each study.

**Table 2:** Number of Inclusion and Exclusion Criteria for the Three Selected Institutional Studies

	aXa		DERENEDIAB	EWING 2008	Total
	Case	Control			
Inclusion criteria	9	7	10	10	36
Exclusion Criteria	11	5	10	5	31
Total	20	12	20	15	67

The selected studies covered different medical domains described by a variety of terminologies, and included a large range of complex criteria. We chose ongoing studies to be as close as possible of the real life setting of the deployment of a clinical trial.

We extracted the inclusion/exclusion criteria from the local Case Report Form. These three studies include a total of 67 eligibility criteria which are listed in Additional file 1.

### Workflow

#### A/ Normalizing eligibility criteria

After collecting the eligibility criteria from the studies of interest, we divided the normalization process of free-text eligibility criteria to computable criteria into six successive steps performed by a physician expert in medical informatics (YG) (Table 3):

**Table 3:** Description and Examples of the Normalization Process of the Free-text Eligibility Criteria

Normalizing eligibility criteria in 6 steps	Before normalization process	After normalization process
1- *Remove redundancy among inclusion and exclusion criteria*	**IC:** no contraindication to low weight molecular heparin **EC:** any contraindication to low weight molecular heparin	**EC:** any contraindication to low weight molecular heparin
*2- Identify individual criteria (*e.g. *split a single complex criterion into multiple simple criteria)*	**IC:** renal artery imaging dated less than 1 year confirming the existence of two normal-sized kidneys > 90 mm and showing no renal artery stenosis	**IC:** renal artery imaging dated less than 1 year; **IC:** kidney size > 90 mm; **IC:** no renal artery stenosis
3- *Reformulate criteria (*e.g. *negate and switch from inclusion to exclusion)*	**IC:** no renal artery stenosis	**EC:** renal artery stenosis *(Negation removed)*
4- *Recognition of individual medical concepts:*	**EC:** known pregnancy or no effective contraception for women of childbearing age or breastfeeding	- Pregnancy - Effective contraception - Age - Breastfeeding
5- *Remove semantic ambiguity (based on knowledge sources or human expertise)*	- Female contraception	- Oral contraception - Intrauterine device - Diaphragm - Spermicide
	- Impossible follow-up	- No translation
6- *Translating medical concepts from local languages to English*	**EC:** Grossesse connue ou absence de contraception efficace pour les femmes en âge de procréer ou allaitement.	**EC:** Known pregnancy or no effective contraception for women of childbearing age or breastfeeding

*IC* inclusion criterion, *EC* exclusion criterion

1. *Remove redundancy among inclusion and exclusion criteria*: some exclusion criteria were only complements of existing inclusion criteria.
2. *Identify individual criteria,* i.e. *split a single complex criterion into multiple simple criteria.*
3. *Reformulate criteria,* e.g. negate and switch from inclusion to exclusion: we transformed inclusion criteria with a negative formulation into exclusion criteria and conversely. This formulation was imposed by the use of the ECLECTIC syntax.
4. *Recognition of individual medical concepts:* the EHR4CR platform uses a set of international terminologies to express eligibility criteria. We manually identified all medical concepts present in the free-text eligibility criteria that could be mapped to any of the EHR4CR terminologies.
5. *Remove semantic ambiguity (based on knowledge sources or human expertise):* some medical concepts were too coarse-grained or too vague to allow useful mapping to our local terminologies. We extended concepts using the UMLS® or human expertise.
  During this process, some criteria were removed as they were too vague (i.e. no consensual definition) or not computable and, therefore, not possible to extend.
6. *Translating medical concepts from local languages to* English: French and German versions of legacy terminologies were used when available. They were translated to English by a trained physician, when necessary, and validated by a fluent English speaker.

#### B/ Expressing eligibility criteria with the EHR4CR platform

1. *Identify medical concepts using the platform terminologies (ICD-10, SNOMED-CT, PathLex, LOINC, ATC): semi-automatic concept recognition*
  We mapped individualized English-translated medical concepts from eligibility criteria to medical concepts from the EHR4CR terminologies using the UMLS Metathesaurus Browser [https://uts.nlm.nih.gov/]. We did not map medical concepts that corresponded to unstructured data in the local EHRs because the EHR4CR platform works only with structured data.
2. *Translate the query into the ECLECTIC model* [19])
  We used the EHR4CR query platform to express the normalized criteria into the *ad-hoc* ECLECTIC syntax. ECLECTIC syntax allows the combination of standardized concepts and Boolean or temporal operators. For instance, we expressed the following exclusion criterion “Known pregnancy or no effective contraception for women of childbearing age or breastfeeding” as follows:
  1. born() at most 18 year before now or
  2. last diagnosis([SNOMED Clinical Terms:5935008, “Oral contraception”])
  3. and not last diagnosis([ICD-10:Z32.1, “Pregnancy confirmed”])
  4. and not last diagnosis([SNOMED Clinical Terms:169750002, “Mother currently breast-feeding”])”

#### C/ Executing the query on the local clinical data warehouse

We mapped medical concepts from the EHR4CR terminologies to local CDW concepts. We performed automatic mapping when the terminologies used in the CDW were the same as those used in the EHR4CR platform (e.g. ICD-10 and ATC terminologies are used by both the HEGP-CDW and the EHR4CR platform). A physician expert in medical informatics (YG) performed other mappings using the browser of i2b2 Query and Analysis tool.

### Outcomes

We defined metrics in order to assess all the components of the workflow described above:

#### A/ Normalization of free-text eligibility criteria

Each eligibility criterion was categorized according to the proposition of *Ross* et al. as either “simple” or “complex” [20]. A criterion was considered to be simple if it could be expressed as a single concept (or the negation of a single concept) or by a simple quantitative comparison. For example, criterion 1 from the aXa study “Age >18”, can be expressed using concept C0001779 from the UMLS associated with a quantitative comparison. The other criteria were considered to be complex.

We calculated the number of criteria per study before and after the 6-step normalization process, the number of unchanged criteria after the normalization process, and the number of final computable criteria i.e. normalized criteria for which at least one medical concept had been mapped to the local terminologies.

#### B/ Expressing eligibility criteria with the EHR4CR platform

We calculated the mapping rate between free-text individualized concepts to EHR4CR terminologies as the number of concepts that could be mapped to one or more concepts of the terminologies used by the EHR4CR platform divided by the total number of concepts individualized in free text criteria.

We asked a PI to assess the agreement between free-text eligibility criteria and computable criteria expressed by ECLECTIC using a five-point Likert scale (5: perfect without any information loss, 4: satisfactory, 3: undecided, 2: not satisfactory, 1: not done).

#### C/ Executing the query on the local clinical data warehouse

We calculated the mapping rate from the medical concepts of EHR4CR terminologies to the locally used terminologies as the number of concepts that could be mapped to one or more concepts of the EHR4CR terminologies divided by the total number of concepts expressed with the EHR4CR terminologies.

##### Coverage

Information in the EHRs could be stored in two different formats: structured and non-structured. Examples of structured data are demographics, diagnosis codes, laboratory test results, and so forth. An example of non-structured data is free-text. The EHR4CR platform dealt only with structured data. We labeled each free-text individualized medical concept as “present and structured” if it corresponded to structured data present in the CDW/EHR; “present and unstructured” if it corresponded to unstructured data, and “missing” if it corresponded to data unavailable in the CDW/EHR.

We assessed *completeness* of the HEGP-CDW for each medical concept individualized in eligibility criteria, i.e. the number of distinct patients for which the information was present in the CDW in 2013 divided by the total number of distinct patients hospitalized at HEGP during the same time period.

### Ethics

This study did not involve human or animal subject and thus does not require any approval from an ethics committee.

## Results

### Normalization of free-text eligibility criteria

Out of a total of 67 eligibility criteria from the three studies, 50/67 (74.6%) were considered to be complex based on the Ross classification. The normalized criteria are presented in Additional file 2. After normalization: the DERENEDIAB study was represented by 20 criteria (10 for inclusion and 10 for exclusion), aXa study cases were represented by 20 criteria (10 for inclusion and 10 for exclusion), aXa controls by 11 criteria (6 for inclusion and 5 for exclusion), and EWING by 15 (10 for inclusion and 5 for exclusion). Thirty-two (47.8%) criteria were modified during the normalization process.

Recognition of individual medical concepts: We identified 114 distinct medical concepts from 67 criteria.

### Expressing and querying eligibility criteria with the EHR4CR platform

a/. *Mapping to the terminologies used by the EHR4CR platform:*
  Of the 114 identified medical concepts, 92 (81%) concepts were mapped to EHR4CR terminologies at the time of this report.
  Twenty-three (20.2%) of these medical concepts corresponded to information present only in non-structured data in HEGP-CDW or UKM-EHR, and were not mapped to any EHR4CR terminologies (e.g. “pulmonary artery”, “dyspnea”, “acute pulmonary heart disease”). Two concepts could not be mapped to any of the EHR4CR terminologies at the time of this report: “active cancer” and “ewing sarcoma”.
  At the end of the process, 51 criteria were computable. All of the computable criteria could be expressed using the ECLECTIC syntax. Of the 67 original criteria, 43 (64,2%) had at least a satisfactory agreement to the original criteria, and the concordance for 23 (34,3%) was considered to be perfect. The agreement was considered to be undecided or not satisfactory for 11 (16,4%) and the translation to computable criteria could not been performed for 13 (19,4%) (Table 4).

**Table 4:** Agreement between Original Criteria and Computed Criteria using a Likert Scale

Likert Scale	aXa		DERENEDIAB N	EWING 2008 N	*Total* N
	Cases N	Controls N			
5: Perfect without information loss	*8*	*4*	*6*	*5*	*23*
4: Satisfactory	*4*	*4*	*6*	*6*	*20*
3: Undecided	*1*	*0*	*0*	*2*	*3*
2: Not satisfactory	*2*	*1*	*5*	*0*	*8*
1: Not done	*5*	*3*	*3*	*2*	*13*
b/. *Mapping medical concepts expressed using EHR4CR terminologies to i2b2 CDW* local terminologies. We mapped 86/92 (93,5%) expressed medical concepts using EHR4CR terminologies to one or more concepts of the local terminologies.
c/. *Coverage of the CDW* (Table 5)

**Table 5:** Results of the normalization process for three institutional studies

Outcomes	aXa		DERENEDIAB	EWING 2008	Total
	Cases	Controls			
ELIGIBILITY CRITERIA					
Criteria **before** normalization	20	12	20	15	67
Complex criteria	15 (75%)	10 (83%)	15 (75%)	10 (67%)	50 (75%)
Unchanged criteria	9 (45%)	8 (67%)	10 (50%)	9 (60%)	36 (54%)
Criteria **after** normalization	20 (100%)	11 (92%)	20 (100%)	15 (100%)	66 (99%)
MEDICAL CONCEPTS					
Individual medical concepts identified in free-text eligibility criteria	45	16	39	14	114
Medical concepts mapped to EHR4CR terminologies^a^	30 (67%)	15 (94%)	38 (97%)	9 (64%)	92 (81%)
Medical concepts mapped from EHR4CR terminologies to local terminologies	30 (67%)	15 (94%)	34 (87%)	7 (50%)	86 (75%)
Free-text medical concepts ***present and structured*** in the local CDW/EHR	30 (67%)	16 (100%)	34 (87%)	9 (64%)	89 (78%)
Free-text medical concepts ***present and unstructured*** in the local CDW/EHR	15 (33%)	0 (0%)	4 (10%)	4 (29%)	23 (20%)
Free-text medical concepts ***missing*** in the local CDW/EHR	0 (0%)	0 (0%)	1 (3%)	1 (7%)	2 (2%)
FINAL COMPUTABLE CRITERIA	14	8	17	12	51

^a^ medical concepts that correspond to unstructured data in local CDW/EHR were not mapped to EHR4CR terminologies
  Twenty-three (20,2%) of the 114 distinct medical concepts corresponded to information present in a non-structured format in the CDW (e.g. “pulmonary artery”, “dyspnea”, “pulmonary heart disease”), and 89 (78,1%) to structured data. Two concepts were considered to be “missing”: “Protein/Creatinine Ratio Urine” and “Lansky score”.
d/. *Completeness*
  Data completeness was assessed in the HEGP-CDW and report for each medical concept that was individualized during the normalization process. The results are reported in Additional file 3.

## Discussion

The re-use of medical data contained in EHRs is a critical issue for clinical research as it could have a major impact on the overall cost of future clinical studies. CTRSSs have been developed to support clinical research at each step of the process of clinical research. The objective of this work is to describe all the necessary tasks for the purpose of re-using the EHR4CR platform at a local level to support existent academic studies. We identified specific issues that could have an impact on the future results of the platform. We also provided metrics to report these critical aspects that could also be used for future reports of CTRSS assessment studies.

The normalization process, i.e. transforming a raw eligibility criterion into a computable criterion, was one of these critical aspects. Most of our final computable criteria (64,2% 43/67) were considered to be at least satisfactory by our expert in clinical research. The choice of working with internationally recognized medical terminologies had an impact on this result. We were able to map 75% of the medical concepts identified in the original criteria to the locally used terminologies from our EHR. These important results highlight the fact that technical specifications of both EHRs and CTRSSs are a major concern for re-using routine care data for clinical research purposes.

### Translating free-text eligibility criteria into computable criteria

#### Terminologies

One of the major limiting aspects of the translation of free-text eligibility criteria into computable criteria is the choice of the terminologies. Most eligibility criteria are expressed in natural language and not as medical concepts. Several options are available. The Unified Medical Language System (UMLS®) is the most complete choice and offers the benefit of its interoperability with many medical terminologies but may be too broad in scope for clinical research. Members of the EHR4CR project decided to work with various international standardized terminologies covering various medical domains. The selected terminologies allowed us to express 81% (92/114) of the medical concepts contained in the original criteria.

There are several benefits for using standards, including, but not limited to, (a) more synonyms available for semi-automated matching, or concept identification, (b) reduced risk of misunderstanding, (c) sharing across recruitment platforms, which would be difficult otherwise.

The choice of a terminology set is important and has a major impact on the number of free-text eligibility criteria that can be transformed into suitable computable criteria.

#### Syntax complexity

Criteria are organized into concepts such as age, gender, disease, symptoms, medical history, and are often connected with comparators including Booleans and others such as “at least”, “more than” or “if then” conditions. This complexity could result in difficulties to express eligibility criteria as computable criteria. The choice of syntax is a second key issue because it affects the ability to translate the eligibility criteria into computable criteria. Weng et al. [21] identified several solutions including (a) re-using the syntax of existing languages such as the Structured Query Language (SQL), (b) developing dedicated syntax e.g. the Arden syntax, or GELLO, (c) logic-based languages, or (d) *ad hoc* solutions [22–24]. The EHR4CR group decided to develop its own solution called ECLECTIC [19]. We expressed all the aXa and DERENEDIAB trial computable criteria using ECLECTIC. This second issue is also crucial because it determines the ability of the platform to express “real world” eligibility criteria and therefore has a direct impact on the final performance.

#### Normalization process

One of the key issues of the normalization process is the criteria complexity. By analyzing 1000 eligibility criteria randomly selected in ClinicalTrials.gov, Ross J. et al. found that 85% contained semantic complexity and that 36,8% were not comprehensible or require clinical judgment or additional metadata [20]. The complexity of our criteria (74.6% (50/67)) comparable to that of other studies.

The pre-processing of free-text eligibility criteria for normalization is necessary for obtaining simple and useful computable criteria. Only 53.7% (36/67) of the criteria did not need any modification to be computed after normalization. This step remains difficult to automate and often requires expert input to remove semantic ambiguity, to extend free-text medical concepts, and for concept recognition. For example, the sixth aXa criterion, containing 14 medical concepts consisting of unstructured data, received a note of 5 “translated without any loss of information” by our clinical research expert. This apparently astonishingly good result was explained by the association of very specific diagnostic conditions (e.g. “**pulmonary embolism confirmed** by a gap in a pulmonary artery” or “**Lower extremity Deep vein thrombosis** confirmed by the lack of compressibility of a vein segment under the ultrasound probe”) combined to non-useful information (in this context) immediately identifiable by an expert. The EHR4CR platform queries EHR billing codes and therefore already works with confirmed diagnoses.

The translation of free-text criteria into medical concepts is a difficult and crucial task. To limit the risk of introducing bias and to better manage subjective topics, complex criteria should be reviewed - or better independently translated - by two or more experts. Moreover, a good comprehension of the content of data warehouse is needed to ensure quality mappings (especially to manage negation in an open world assumption in which the absence of information is not equivalent to the negation)

### Identifying medical concepts using the platform terminologies

The second part of the mapping process consists of connecting the translated computable criteria now translated into biomedical concept from standard terminologies to local EHRs terminologies. Many automated solutions have been proposed but human expertise is still needed to insure high quality mapping. We were able to map 93,5% (86/92) of the medical concepts represented by EHR4CR terminologies to the locally used terminologies. This success is because many of the EHR4CR terminologies were also used in the local CDWs.

While information in EHRs is often structured, mapping concepts of structured eligibility criteria to medical concepts collected in a care context is challenging: (a) terminologies used to translate eligibility criteria are not necessarily the same as those used for storing routine care data in EHRs and (b) there is not always a direct relationship between these different terminologies. For example, “Protein/Creatinine Ratio in urine” would have required a concomitant determination of protein and creatinine in urine and a calculation to be mapped. Similarly, one simple concept could be potentially represented in EHRs in various forms. For example, patients with “Acute Kidney Disease” could be identified in EHRs using diagnosis codes, laboratory results, or free-text. Some studies have developed computer algorithms to identify specific patients with EHR data [25, 26]. Natural language processing tool functionalities of the EHR4CR platform were not available during this work. Therefore, we did not map the medical concepts that corresponded to unstructured data to EHR4CR terminologies. This was possible because the medical informatics experts responsible for the normalization process had a perfect knowledge of the CDW contents.

### Structured data completeness and missing data of EHRs to support clinical research

Structured data are the easiest to use in the implementation of a CTRSS but these data are collected for purposes other than patient recruitment into clinical trials. Nevertheless, we found that 78% (89/114) of the medical concepts present in the aXa, DERENEDIAB and EWING 2008 criteria corresponded to structured data. This is more than the 55% identified by Köpcke et al. who worked on the EHRs from five German University Hospitals [27]. This may be because the definition of medical concepts in our study was not exactly the same as in theirs resulting in differences between the results of the two studies. Future studies aiming to assess CTRSS performance need to specify the data completeness for each medical concept to provide comparable outcomes.

Missing data is another crucial point that must be carefully addressed during the normalization process. Missing data will influence the results of the platform and this influence will be highly variable depending on if these missing values concern the inclusion or the exclusion criteria. Indeed, eligibility criteria with a high prevalence of missing data decrease the sensitivity of the platform because all eligibility criteria are associated with “AND” operators in the platform. For example, the use of contraception or not is is far from being reported for all women in the HEGP-CDW. Eligibility criteria containing this concept decrease the number of identified patients. Missing data also increase the number of patients falsely identified and therefore the specificity of the platform because all exclusion criteria are associated with “AND NOT” operators. Another important issue is that missing data are not objectively quantifiable in a CDW. Thus, local expertise from people with a complete knowledge of the queried CDW and a perfect understanding of the objectives of the future supported trials are absolutely necessary before using the platform.

Completeness was evaluated for the entire data warehouse. Such an evaluation is relevant for items such as gender. Low completeness for a given criterion may exclude participation of a medical center. However, for specific results, completeness is only relevant for a subset of patients for whom the information is needed (e.g. HbA1c for diabetic patients).

### Time issues

Another critical issue from the point of view of the CTRSS user is the question of time. Each eligibility criterion may vary over time: new biological measurements, drug therapies, diagnoses, etc. The frequency with which the database is queried will have an influence on the results and especially on the sensitivity of the CTRSS. In most cases, data contained in CDWs are not real-time data. For example, to be collected in our CDW, diagnoses must be 1/ coded by the physician and 2/ imported. Any real-time inclusion study (e.g. inclusion at diagnosis or immediately after) is problematic, especially for acute diseases.

### Feasibility of the approach

The process described in this study is time consuming. In our case, the mapping and translation of the criteria was performed successfully by an expert physician experienced in medical informatics and with a strong knowledge of the content and structure of the local clinical data warehouse. If the platform such as EHR4CR were to be developed, specialized support team composed of medical informatics specialists and data-manager would probably be needed to enable scalability.

### General statements about the EHR4CR normalization (or standardization) pipeline

- Semantic interoperability is one of the main challenges to address to enable the reuse of hospital EHR data to support research. Semantic interoperability within a broad international research network reusing clinical data from EHRs requires a rigorous governance process to ensure the quality of the data standardization process.
- This study demonstrates good coverage of the EHR4CR central terminology used during the normalization process of the eligibility criteria of three studies. However, its scope needs to be continuously extended to address the representation of a much broader set of eligibility criteria. Updating of the EHR4CR central terminology cannot be fully automated (e.g. through automatic coding of free text clinical trial protocols). A collaborative editor is required to efficiently support the creation of new semantic resources to expand the scope to additional studies and associated eligibility criteria.
- Despite recent efforts, formal representation of multimodal and multi-level data that supports data interoperability across clinical research and care domains is still challenging.
- Terminology mapping at hospital sites is the major bottleneck of the data standardization pipeline. Supportive tools are still in their infancy.

## Conclusion

Only a limited number of studies have assessed the effectiveness of CTRSSs with respect to their sensitivity, specificity and impact on recruitment rate [7]. By trying to re-use the EHR4CR platform for three local institutional studies, we identified a set of issues that may affect the future performance of the platform. These must be specified in future reporting of the assessment of CTRSS accuracy. The recommendations of Köpcke et al. for future reporting of CTRSS performance is based on the literature. In contrast, our recommendations are based on our practical experience in deploying institutional studies (Table 6). We aim to provide strengthened recommendations for future CTRSS reporting that will insure (i) the exact reproducibility of the inclusion/exclusion criteria execution, (ii) a fair comparison of the query execution results, and hence of the platforms themselves. Additional work by domain experts is still needed to harmonize these efforts.

**Table 6:** Recommendations: Informations that Should be Specified in Future CTRSS Assessment Reporting from the CTRSS User Point of View

Normalizing Eligibility Criteria
1- The number of free-text eligibility criteria, and their complexity; 2- The terminologies and syntax that have been used to represent eligibility criteria; 3- The concordance between the original free-text eligibility criteria and the final computable criteria; 4- The quality of the mapping, i.e., the proportion of concepts that have been successfully mapped; 5- If human expertise is necessary at each step of the process and how it could affect the results;
Time Issue
6- How often the EHR has been queried and for what period of time;
Structured Data Completeness of EHR
7- The completeness of the database(s) for each eligibility criterion, i.e. is the data available and for what proportion of patients? 8- If the platform was used to query structured data, unstructured data or both; 9- How the issue of data completeness has been managed: not managed, with scoring, Bayesian network, free text mining using natural language processing tools…

## Additional files

Additional file 1: Free Text Eligibility Criteria for DERENEDIAB, aXa and EWING 2008 studies (DOCX 202 kb)
Additional file 2: Normalized Criteria for the DERENEDIAB, aXa and EWING 2008 studies (DOCX 49 kb)
Additional file 3: HEGP-CDW Data Completeness for each Medical Concept Individualized During the Normalization Process (DOCX 28 kb)

## Abbreviations

CDW: Clinical data warehouse
CTRSS: Clinical Trial Recruitment Support Systems
EFPIA: European Federation of Pharmaceutical Industries and Associations
EHR: Electronic Health Records
EHR4CR: Electronic records for clinical research
ETL: Extract transform load
SQL: Structured Query Language
UMLS: Unified Medical Language System
WWU: Westfälische Wilhelms-Universität Münster
