# Supplementary material for: Leveraging the EHR4CR platform to support patient inclusion in academic studies: challenges and lessons learned
Source: BMC Med Res Methodol. 2017 Feb 28;17:36. doi: 10.1186/s12874-017-0299-3 (PMC5329914; doi:10.1186/s12874-017-0299-3)
Supplement: Additional file 1: — Free Text Eligibility Criteria for DERENEDIAB, aXa and EWING 2008 studies (DOCX 202 kb) [file 12874_2017_299_MOESM1_ESM.docx]

**Additional File 1: Free Text Eligibility Criteria for DERENEDIAB, aXa and EWING 2008 studies**

| Study | IC/EC | N | Criterion |
| --- | --- | --- | --- |
| DERENEDIAB | IC | 1 | d’âge ≥18 et ≤75 ans |
| DERENEDIAB | IC | 2 | patients diabétiques de type 2 |
| DERENEDIAB | IC | 3 | des 2 sexes. Les femmes doivent être ménopausées depuis un an, ou être stériles secondairement à une chirurgie, ou utiliser une méthode contraceptive efficace telle qu'un contraceptif oral, un contraceptif local avec un spermicide ou un dispositif intra-utérin |
| DERENEDIAB | IC | 4 | ayant une néphropathie diabétique (histologiquement prouvée ou non, et dans ce cas on se basera sur la notion de l’association diabète, rétinopathie diabétique, absence d’hématurie) |
| DERENEDIAB | IC | 5 | avec eDFG (MDRD) > 20 ml/min/1,73 m² |
| DERENEDIAB | IC | 6 | avec persistance d’un rapport PU/CrU > 0,1 g/mmol, depuis 8 semaines |
| DERENEDIAB | IC | 7 | sous traitement comportant - depuis au moins 2 mois - au moins 1 bloqueur du SRAA (un IEC à doses usuelles ou un IDR, et/ou un ARAII à doses usuelles) et un diurétique |
| DERENEDIAB | IC | 8 | sans antécédent d’angioplastie et/ou stenting de(s) l’artère(s) rénale(s) |
| DERENEDIAB | IC | 9 | avec imagerie des artères rénales datant de moins d’un an confirmant l’existence de 2 reins de taille normale > 90 mm et ne montrant pas de sténose artérielle rénale |
| DERENEDIAB | IC | 10 | inscrits à un régime de sécurité sociale |
| DERENEDIAB | EC | 1 | Patients trop éloignés géographiquement du lieu de suivi et de traitement |
| DERENEDIAB | EC | 2 | Hypertension artérielle sévère (grade 3 de la classification de l'ESH ; PAS clinique ≥ 180 mmHg et/ou PAD clinique ≥ 110 mmHg) |
| DERENEDIAB | EC | 3 | Pathologie rénale non secondaire au diabète |
| DERENEDIAB | EC | 4 | Allergie sévère à un produit de contraste iodé (choc, œdème de Quincke ) |
| DERENEDIAB | EC | 5 | Antécédent de fibrose néphrogénique aux produits de contraste utilisés en IRM |
| DERENEDIAB | EC | 6 | Pace-maker ou autre objet métallique, claustrophobie contrindiquant l’utilisation de l’IRM |
| DERENEDIAB | EC | 7 | Pace-maker et/ou défibrillateurs implantables contrindiquant la délivrance d’ondes radio basse fréquence |
| DERENEDIAB | EC | 8 | Pathologie aorto-iliaque sévère et/ou antécédents de maladie des emboles de cholestéro |
| DERENEDIAB | EC | 9 | Anatomie rénale défavorable pour la DR (notion de rein unique, atrophie rénale, artères rénales multiples) |
| DERENEDIAB | EC | 10 | Prothèse aortique mise en place par voie chirurgicale ou par voie endovasculaire |
| aXa/Case | IC | 1 | Etre âgé de plus de 18 ans |
| aXa/Case | IC | 2 | Etre affilié à un régime de sécurité sociale ou ayant droit |
| aXa/Case | IC | 3 | Etre atteint de cancer solide évolutif ou hématologique évolutif (myélome ou lymphome), confirmé histologiquement ou cytologiquement, dont le caractère évolutif sera défini par l’existence d’une maladie tumorale active ou résection tumorale incomplète ou marqueurs tumoraux restés élevés après résection complète |
| aXa/Case | IC |  | La maladie veineuse thromboembolique est : |
| aXa/Case | IC | 4 | soit une thrombose veineuse profonde des membres inférieurs (proximale ou distale) confirmée soit par l’absence de compressibilité d’un segment veineux sous la sonde d’échographie, soit par la présence d’une lacune veineuse sur le phléboscanner ou la phlébographie |
| aXa/Case | IC | 5 | soit une thrombose iliaque ou cave objectivée par un scanner abdominal injecté ou par échographie veineuse ou par iliocavographie, |
| aXa/Case | IC | 6 | soit une embolie pulmonaire confirmée objectivement selon les critères des recommandations de la société Européenne de cardiologie [17] : (1) par une lacune dans une artère pulmonaire, au moins segmentaire ou des lacunes multiples sous-segmentaires sur un angioscanner spiralé des artères pulmonaires ou (2) par un aspect de haute probabilité sur une scintigraphie de ventilation-perfusion, ou (3) par des symptômes cliniques d’embolie pulmonaire accompagnant une thrombose veineuse proximale symptomatique confirmée par échographie veineuse ou (4) par un coeur pulmonaire aigu échocardiographique inexpliqué en présence d’une forte probabilité clinique chez un patient en état de choc cardiogénique intransportable. |
| aXa/Case | IC | 7 | La maladie thromboembolique peut être symptomatique ou découverte de façon fortuite mais est confirmée objectivement |
| aXa/Case | IC | 8 | Absence de contre-indication à un traitement par héparine de bas poids moléculaire à dose thérapeutique. |
| aXa/Case | IC | 9 | Prescription depuis moins de 72h d’un traitement par héparine de bas poids moléculaire ou fondaparinux à dose thérapeutique. |
| aXa/Case | EC | 1 | Thrombose veineuse viscérale, du membre supérieur ou thrombose veineuse du système cave supérieur, car leur évolutivité sous traitement, notamment le risque de récidive embolique est moins bien connu que celui des embolies pulmonaires et des thromboses des membres inférieurs et que leurs modalités diagnostiques sont moins bien formalisées |
| aXa/Case | EC | 2 | Maladie tumorale non confirmée histologiquement ou cytologiquement |
| aXa/Case | EC | 3 | Suivi après résection tumorale complète sans élévation des marqueurs tumoraux |
| aXa/Case | EC | 4 | Contre-indication au traitement curatif par héparine de bas poids moléculaire |
| aXa/Case | EC | 5 | Traitement initial par une autre molécule anticoagulante autre qu’une HBPM ou du fondaparinux (antithrombine directe, inactivateur direct du facteur Xa) |
| aXa/Case | EC | 6 | Insuffisance rénale sévère définie par une clairance à la créatinine inférieure à 30 ml/min à l’inclusion |
| aXa/Case | EC | 7 | Grossesse connue ou allaitement |
| aXa/Case | EC | 8 | Patient préalablement inclus dans l’étude |
| aXa/Case | EC | 9 | Suivi impossible |
| aXa/Case | EC | 10 | Espérance de vie < 6 mois |
| aXa/Case | EC | 11 | Patient dont le poids est supérieur à 100 Kg |
| aXa/Control | IC | 1 | Etre âgé de plus de 18 ans |
| aXa/Control | IC | 2 | Etre affilié à un régime de sécurité sociale ou ayant droit |
| aXa/Control | IC | 3 | Etre indemne de pathologie tumorale maligne décelable à l’inclusion |
| aXa/Control | IC | 4 | Etre atteint de maladie veineuse thromboembolique définie par les mêmes critères que celle des sujets atteints de cancer |
| aXa/Control | IC | 5 | La maladie thromboembolique peut être symptomatique ou découverte de façon fortuite mais confirmée objectivement |
| aXa/Control | IC | 6 | Absence de contre-indication au traitement par héparine de bas poids moléculaire à dose thérapeutique |
| aXa/Control | IC | 7 | Prescription depuis moins de 72h d’un traitement par héparine de bas poids moléculaire ou fondaparinux à dose thérapeutique |
| aXa/Control | EC | 1 | Suspicion non encore confirmée de pathologie tumorale maligne associée à la maladie veineuse thromboembolique |
| aXa/Control | EC | 2 | Cancer actif datant de moins de 2 ans |
| aXa/Control | EC | 3 | Contre-indication au traitement curatif par héparine de bas poids moléculaire |
| aXa/Control | EC | 4 | Traitement initial par une molécule anticoagulante autre qu’une HBPM ou du fondaparinux (antithrombine directe, inactivateur direct du facteur Xa) |
| aXa/Control | EC | 5 | Insuffisance rénale définie par une clairance à la créatinine inférieure à 30 ml/min à l’inclusion |
| EWING 2008 | IC | 1 | Diagnosis: Histologically confirmed Ewing sarcoma of bone or soft tissue. |
| EWING 2008 | IC | 2 | Age and sex: Either sex, age >48 months (for GPOH patients) and <50 years at the date of diagnostic biopsy. Younger or elderly patients may be reported to the appropriate office (see section 1.4) but are not included in this study. |
| EWING 2008 | IC | 3 | Registration: ≤ 45 days after diagnostic biopsy/surgery. |
| EWING 2008 | IC | 4 | Start of chemotherapy: ≤ 45 days after diagnostic biopsy/surgery. |
| EWING 2008 | IC | 5 | Informed consent: Must be signed prior to study entry. |
| EWING 2008 | IC | 6 | Performance status: Lansky or Karnofsky score > 50%, may be modified for handicapped patients. |
| EWING 2008 | IC | 7 | Haemoglobin > 8 g/dl (transfusion allowed), |
| EWING 2008 | IC | 8 | Platelets > 80.000/µl (transfusion allowed), |
| EWING 2008 | IC | 9 | WBC > 2000/µl. |
| EWING 2008 | IC | 10 | Cardiac values: LVEF > 40%, SF > 28%. |
| EWING 2008 | EC | 1 | More than one cycle of other chemotherapy prior to registration |
| EWING 2008 | EC | 2 | Second malignancy |
| EWING 2008 | EC | 3 | Pregnancy and lactation |
| EWING 2008 | EC | 4 | Concurrent treatment within any other clinical trial, except trials with different endpoints that due to the nature of their endpoints must run parallel to EWING 2008 e.g. trials on antiemetics, antimycotics, antibiotics, strategies for psychosocial support, etc... |
| EWING 2008 | EC | 5 | Any other medical, psychiatric, or social condition incompatible with protocol treatment |
