# Supplementary material for: Leveraging the EHR4CR platform to support patient inclusion in academic studies: challenges and lessons learned
Source: BMC Med Res Methodol. 2017 Feb 28;17:36. doi: 10.1186/s12874-017-0299-3 (PMC5329914; doi:10.1186/s12874-017-0299-3)
Supplement: Additional file 2: — Normalized Criteria for the DERENEDIAB, aXa and EWING 2008 studies (DOCX 49 kb) [file 12874_2017_299_MOESM2_ESM.docx]

Additional File 2: Normalized Criteria for the DERENEDIAB, aXa and EWING 2008 studies

| **Study** | **N** | **Type of criterion** | **Criterion** | **Criterion (English version)** | **Medical concept** |
| --- | --- | --- | --- | --- | --- |
| DERENEDIAB | 1 | IC | d’âge ≥18 et ≤75 ans | Individual is > 18 and ≤ 75 years old | Age |
| DERENEDIAB | 2 | IC | patients diabétiques de type 2 | Type 2 diabetes mellitus male or female patient | Type 2 diabetes mellitus |
| DERENEDIAB | 3 | IC | des 2 sexes. Les femmes doivent être ménopausées depuis un an, ou être stériles secondairement à une chirurgie, ou utiliser une méthode contraceptive efficace telle qu'un contraceptif oral, un contraceptif local avec un spermicide ou un dispositif intra-utérin | From both genders. Women have to be postmenopausal for a year, or be sterile after surgery, or use an effective contraceptive method such as an oral contraceptive, a local contraceptive with a spermicide or an intrauterine device | Male |
|  |  |  |  |  | Female |
|  |  |  |  |  | Menopause |
|  |  |  |  |  | Female Infertility |
|  |  |  |  |  | Hysterectomy (procedure) |
|  |  |  |  |  | Bilateral tubal ligation (procedure) |
|  |  |  |  |  | Oral contraception |
|  |  |  |  |  | Intrauterine device |
|  |  |  |  |  | Diaphragm |
|  |  |  |  |  | Spermicide |
| DERENEDIAB | 4 | IC | ayant une néphropathie diabétique (histologiquement prouvée ou non, et dans ce cas on se basera sur la notion de l’association diabète, rétinopathie diabétique, absence d’hématurie) | Diabetic nephropathy (if no pathological examination, diagnosis based on the association of history of diabetes, diabetic retinopathy and no hematuria) | Diabetic Nephropathy |
|  |  | IC |  |  | Diabetic retinopathy |
|  |  | IC |  |  | hematuria |
| DERENEDIAB | 5 | IC | avec eDFG (MDRD) > 20 ml/min/1,73 m² | with eDFG (MDRD) > 20 ml/min/1,73 m² | Creatinine clearance rate |
| DERENEDIAB | 6 | IC | avec persistance d’un rapport PU/CrU > 0,1 g/mmol, depuis 8 semaines | Proteinuria/creatininuria ratio > 0.1 g/mmol lasting for 8 weeks | Protein/Creatinine Ratio |
| DERENEDIAB | 7 | IC | sous traitement comportant - depuis au moins 2 mois - au moins 1 bloqueur du SRAA (un IEC à doses usuelles ou un IDR, et/ou un ARAII à doses usuelles) et un diurétique | Under stable medication regimen including for at least 2 months full tolerated doses of al least 1 RAAS blocker (ACEI, renin inhibitor, ARB) and a diuretic | Angiotensin-converting-enzyme inhibitors |
|  |  |  |  |  |  |
|  |  |  |  |  | Angiotensin II receptor antagonists |
|  |  |  |  |  | Diuretics |
| DERENEDIAB | 8 | IC | avec imagerie des artères rénales datant de moins d’un an confirmant l’existence de 2 reins de taille normale > 90 mm et ne montrant pas de sténose artérielle rénale | With imaging of renal arteries dating less than one year confirming the existence of 2 kidneys of normal size> 90 mm and showing no renal artery stenosis | Renal artery ultrasonography, doppler |
|  |  |  |  |  | Renal artery spiral computed tomography angiography |
| DERENEDIAB | 9 | IC | inscrits à un régime de sécurité sociale | Health insurance policy active |  |
| DERENEDIAB | 1 | EC | Patients trop éloignés géographiquement du lieu de suivi et de traitement | Patients living too far from the specialized center | _ |
| DERENEDIAB | 2 | EC | Hypertension artérielle sévère (grade 3 de la classification de l'ESH ; PAS clinique ≥ 180 mmHg et/ou PAD clinique ≥ 110 mmHg) | Patients with severe hypertension (grade 3 ESH classification) ; PAS ≥ 180 mmHg and/or PAD clinique ≥ 110 mmHg) | Systolic pressure |
|  |  |  |  |  | Diastolic pressure |
| DERENEDIAB | 3 | EC | Allergie sévère à un produit de contraste iodé (choc, œdème de Quincke ) | Patient with contrast media allergy | Iodine allergy |
| DERENEDIAB | 4 | EC | Antécédent de fibrose néphrogénique aux produits de contraste utilisés en IRM | Medical history of Nephrogenic Systemic Fibrosis caused by MRI contrast agents | Nephrogenic fibrosis |
|  |  |  |  |  | Magnetic resonance imaging contrast media |
| DERENEDIAB | 5 | EC | Claustrophobie contrindiquant l’utilisation de l’IRM | Claustrophobia contraindicating the use of MRI | Specific (isolated) phobias |
| DERENEDIAB | 6 | EC | Pace-maker et/ou défibrillateurs implantables contrindiquant la délivrance d’ondes radio basse fréquence | Patient with any implantable device incompatible with low frequency waves delivery | Pacemaker |
|  |  |  |  |  | Defibrillators, implantable |
| DERENEDIAB | 7 | EC | Pathologie aorto-iliaque sévère et/ou antécédents de maladie des emboles de cholestérol | Severe aortoiliac disease and/or medical history of cholesterol embolism syndrome | cholesterol embolism syndrome |
|  |  |  |  |  | Aortic aneurysm and dissection |
|  |  |  |  |  | Aneurysm of iliac artery |
|  |  |  |  |  | Embolism and thrombosis of abdominal aorta |
|  |  |  |  |  | Embolism and thrombosis of iliac artery |
| DERENEDIAB | 8 | EC | Anatomie rénale défavorable pour la DR (notion de rein unique, atrophie rénale, artères rénales multiples) | Unfavorable renal anatomy for the renal denervation (solitary kidney, renal agenesis, multiple renal arteries) | Renal agenesis and other reduction defects of kidney |
|  |  |  |  |  | Unspecified contracted kidney |
|  |  |  |  |  | Other congenital malformations of renal artery |
| DERENEDIAB | 9 | EC | Prothèse aortique mise en place par voie chirurgicale ou par voie endovasculaire | Aortic prosthesis implanted by surgical or endovascular procedure | Procedure on aorta (procedure) |
| DERENEDIAB | 10 | EC | antécédent d’angioplastie et/ou stenting de(s) l’artère(s) rénale(s) | Medical history of renal artery angioplasty and/or stenting | Renal artery angioplasty |
|  |  |  |  |  | Renal artery stenting |
| DERENEDIAB | 11 | EC | sténose artérielle rénale | sténose artérielle rénale | Atherosclerosis of renal artery |
| aXa Case | 1 | IC | Etre âgé de plus de 18 ans | 18 Years and older | age |
| aXa Case | 2 | IC | Etre affilié à un régime de sécurité sociale ou ayant droit | Being affiliated to a social security scheme | _ |
| aXa Case | 3 | IC | Etre atteint de cancer solide évolutif ou hématologique évolutif (myélome ou lymphome), confirmé histologiquement ou cytologiquement, dont le caractère évolutif sera défini par l’existence d’une maladie tumorale active ou résection tumorale incomplète ou marqueurs tumoraux restés élevés après résection complète | Having an active solid or hematological cancer (myeloma or lymphoma), histologically or cytologically confirmed, for which the active state will be defined by the existence of a tumoral active disease or an incomplete tumoral resection or the persistence of high tumor markers after complete resection of the tumor. | Malignant neoplasm |
|  |  |  |  |  | Neoplasms of uncertain or unknown behaviour |
|  |  |  |  |  | antineoplastic agents |
|  |  |  |  |  | Radiotherapy |
|  |  |  | La maladie veineuse thromboembolique est : | The venous thromboembolism disease has to be: |  |
| aXa Case | 4 | IC | soit une thrombose veineuse profonde des membres inférieurs (proximale ou distale) confirmée soit par l’absence de compressibilité d’un segment veineux sous la sonde d’échographie, soit par la présence d’une lacune veineuse sur le phléboscanner ou la phlébographie | A deep vein thrombosis of lower extremity (proximal or distal) confirmed by the lack of compressibility of a venous segment under the ultrasound probe or the presence of a venous gap in CT venography or phlebography; | Phlebitis and thrombophlebitis of femoral vein |
|  |  |  |  |  | Phlebitis and thrombophlebitis of other deep vessels of lower extremities |
|  |  |  |  |  | Phlebitis and thrombophlebitis of lower extremities, unspecified |
| aXa Case | 5 | IC | soit une thrombose iliaque ou cave objectivée par un scanner abdominal injecté ou par échographie veineuse ou par iliocavographie, | or a thrombosis of the vena cava or the iliac vein confirmed by an abdominal CT scan with contrast or a venous ultrasound or an ilio-cavography | Embolism and thrombosis of vena cava |
|  |  |  |  |  | Iliac vein thrombosis |
| aXa Case | 6 | IC | soit une embolie pulmonaire confirmée objectivement selon les critères des recommandations de la société Européenne de cardiologie [17] : (1) par une lacune dans une artère pulmonaire, au moins segmentaire ou des lacunes multiples sous-segmentaires sur un angioscanner spiralé des artères pulmonaires ou (2) par un aspect de haute probabilité sur une scintigraphie de ventilation-perfusion, ou (3) par des symptômes cliniques d’embolie pulmonaire accompagnant une thrombose veineuse proximale symptomatique confirmée par échographie veineuse ou (4) par un coeur pulmonaire aigu échocardiographique inexpliqué en présence d’une forte probabilité clinique chez un patient en état de choc cardiogénique intransportable. | or a pulmonary embolism confirmed according to the guidelines of the European Society of Cardiology : through a gap in a pulmonary artery, at least segmental or multiple gaps sub-segmental on spiral CT angiography of the pulmonary arteries or by a high appearance probability on a lung radionuclide imaging, or by clinical symptoms of pulmonary embolism accompanying symptomatic proximal vein thrombosis confirmed by a venous ultrasound or by an unexplained echocardiography acute pulmonary heart in presence of a high clinical probability and for patients who are unfit for transport and with cardiogenic shock | Pulmonary embolism |
| aXa Case | 7 | IC | La maladie thromboembolique peut être symptomatique ou découverte de façon fortuite mais est confirmée objectivement | The venous thromboembolism disease can be asymptomatic or incidentally discovered but is confirmed objectively. | _ |
| aXa Case | 8 | IC | Prescription depuis moins de 72h d’un traitement par héparine de bas poids moléculaire ou fondaparinux à dose thérapeutique. | Prescription in the last 72 hours of a low molecular weight heparin treatment or fondaparinux at therapeutic dose. | Dalteparin |
|  |  |  |  |  | Enoxaparin |
|  |  |  |  |  | Nadroparin |
|  |  |  |  |  | Reviparin |
|  |  |  |  |  | Tinzaparin |
|  |  |  |  |  | Fondaparinux |
| aXa Case | 9 | IC | "contraception efficace pour les femmes en age procréer" | “lack of effective contraception for women of childbearing age “ | Oral contraception |
|  |  |  |  |  | Intrauterine device |
|  |  |  |  |  | Diaphragm |
|  |  |  |  |  | Spermicide |
| aXa Case | 10 | IC | "Maladie tumorale confirmée histologiquement ou cytologiquement" | “Tumor disease histologically or cytologically confirmed“ | Histological |
|  |  |  |  |  | Cytological |
| aXa Case | 1 | EC | Thrombose veineuse viscérale, du membre supérieur ou thrombose veineuse du système cave supérieur, car leur évolutivité sous traitement, notamment le risque de récidive embolique est moins bien connu que celui des embolies pulmonaires et des thromboses des membres inférieurs et que leurs modalités diagnostiques sont moins bien formalisées | Visceral vein thrombosis of the upper limb or venous thrombosis of the superior vena cava system because their scalability under treatment, including the risk of embolic recurrence is less known that pulmonary embolism and thrombosis of the lower limbs and their diagnostic modalities are less formalized. | Thrombosis of cardiac and vascular prosthetic devices, implants and grafts |
|  |  |  |  |  | Cerebral venous thrombosis in the puerperium |
|  |  |  |  |  | Cerebral venous thrombosis in pregnancy |
|  |  |  |  |  | Phlebitis of portal vein |
|  |  |  |  |  | Embolism and thrombosis of renal vein |
|  |  |  |  |  | Budd-Chiari syndrome |
|  |  |  |  |  | Portal vein thrombosis |
|  |  |  |  |  | Nonpyogenic thrombosis of intracranial venous system) |
|  |  |  |  |  | Cerebral infarction due to cerebral venous thrombosis, nonpyogenic |
|  |  |  |  |  | Intracardiac thrombosis, not elsewhere classified |
|  |  |  |  |  | Heparin induced thrombocytopenia (HIT) |
| aXa Case | 2 | EC | Suivi après résection tumorale complète sans élévation des marqueurs tumoraux | Follow-up after complete tumor resection without elevated tumor markers | _ |
| aXa Case | 3 | EC | Contre-indication au traitement curatif par héparine de bas poids moléculaire | Cons-indication to low molecular weight heparin treatment at therapeutic dose | Nontraumatic subarachnoid hemorrhage from middle cerebral artery |
|  |  |  |  |  | Nontraumatic subarachnoid hemorrhage from unspecified intracranial artery |
|  |  |  |  |  | Nontraumatic subarachnoid hemorrhage, unspecified |
|  |  |  |  |  | Nontraumatic intracerebral hemorrhage, intraventricular |
|  |  |  |  |  | Nontraumatic intracerebral hemorrhage, unspecified |
|  |  |  |  |  | Nontraumatic intracerebral hemorrhage, intraventricular |
|  |  |  |  |  | Nontraumatic extradural hemorrhage |
|  |  |  |  |  | Focal traumatic brain injury |
|  |  |  |  |  | Traumatic subdural hemorrhage |
|  |  |  |  |  | Traumatic subarachnoid hemorrhage |
|  |  |  |  |  | Gastrointestinal haemorrhage, unspecified |
|  |  |  |  |  | Other specified intracranial injuries |
|  |  |  |  |  | Other nontraumatic intracranial haemorrhage |
|  |  |  |  |  | Intracerebral haemorrhage |
|  |  |  |  |  | Coagulation defects, purpura and other haemorrhagic conditions |
|  |  |  |  |  | Haematemesis |
|  |  |  |  |  | Esophageal varices with bleeding |
|  |  |  |  |  | Secondary esophageal varices with bleeding |
|  |  |  |  |  | Ulcer of esophagus with bleeding |
|  |  |  |  |  | Melena |
|  |  |  |  |  | Chronic or unspecified peptic ulcer, site unspecified, with hemorrhage |
|  |  |  |  |  | Chronic or unspecified peptic ulcer, site unspecified, with both hemorrhage and perforation |
| aXa Case | 4 | EC | Traitement initial par une autre molécule anticoagulante autre qu’une HBPM ou du fondaparinux (antithrombine directe, inactivateur direct du facteur Xa) | Initial treatment with another anticoagulant molecule than LMWH or fondaparinux (thrombin inhibitor, direct factor Xa inhibitors) | thrombin inhibitors |
|  |  |  |  |  | Dabigatran etexilate |
|  |  |  |  |  | Argatroban |
|  |  |  |  |  | Rivaroxaban |
|  |  |  |  |  | Apixaban |
| aXa Case | 5 | EC | Insuffisance rénale sévère définie par une clairance à la créatinine inférieure à 30 ml/min à l’inclusion | Severe renal impairment defined by a creatinine clearance below than 30 ml / min at baseline | Creatinine clearance rate |
| aXa Case | 6 | EC | Grossesse connue ou absence de contraception efficace pour les femmes en âge de procréer ou allaitement | Known pregnancy or lack of effective contraception for women of childbearing age or breastfeeding | Pregnancy |
|  |  |  |  |  | Breast Feading |
| aXa Case | 7 | EC | Patient préalablement inclus dans l’étude | Patient previously included in the study | _ |
| aXa Case | 8 | EC | Suivi impossible | Impossible follow-up | _ |
| aXa Case | 9 | EC | Espérance de vie < 6 mois | Life expectancy less than 6 months | _ |
| aXa Case | 10 | EC | Patient dont le poids est supérieur à 100 Kg | Patient whose weight is greater than 100 Kg | Poids |
| EWING 2008 | 1 | IC | Diagnosis: Histologically confirmed Ewing sarcoma of bone or soft tissue. | Diagnosis: Histologically confirmed Ewing sarcoma of bone or soft tissue. | Diagnosis/Text: Ewing sarcoma |
| EWING 2008 | 2 | IC | Age and sex: Either sex, age >48 months (for GPOH patients) and <50 years at the date of diagnostic biopsy. Younger or elderly patients may be reported to the appropriate office (see section 1.4) but are not included in this study. | Age and sex: Either sex, age >48 months (for GPOH patients) and <50 years at the date of diagnostic biopsy. Younger or elderly patients may be reported to the appropriate office (see section 1.4) but are not included in this study. | Gender: male or female |
| EWING 2008 | 3 | IC | Registration (<=45 days after biopsy or surgery) | Registration (<=45 days after biopsy or surgery) | Registration (<=45 days after biopsy or surgery) |
| EWING 2008 | 4 | IC | Procedure: chemotherapy (<=45 days after biopsy or surgery) | Procedure: chemotherapy (<=45 days after biopsy or surgery) | Procedure: chemotherapy (<=45 days after biopsy or surgery) |
| EWING 2008 | 5 | IC | Informed consent | Informed consent | Informed consent |
| EWING 2008 | 6 | IC | Score/Lansky: >50% | Score/Lansky: >50% | Score/Lansky: >50% |
| EWING 2008 | 7 | IC | Lab.finding/Hemoglobin: >8g/dl | Lab.finding/Hemoglobin: >8g/dl | Lab.finding/Hemoglobin: >8g/dl |
| EWING 2008 | 8 | IC | Lab.finding/Platelets: >80000/µl | Lab.finding/Platelets: >80000/µl | Lab.finding/Platelets: >80000/µl |
| EWING 2008 | 9 | IC | Lab.finding/WBC: >2000/µl | Lab.finding/WBC: >2000/µl | Lab.finding/WBC: >2000/µl |
| EWING 2008 | 10 | IC | Finding/LVEF: >40% | Finding/LVEF: >40% | Finding/LVEF: >40% |
| EWING 2008 | 1 | EC | Procedure:Chemotherapy (>1x) | Procedure:Chemotherapy (>1x) | Procedure:Chemotherapy (>1x) |
| EWING 2008 | 2 | EC | Diagnosis: any other malignancy | Diagnosis: any other malignancy | Diagnosis: any other malignancy |
| EWING 2008 | 3 | EC | Pragnancy | Pragnancy | Pragnancy |
| EWING 2008 | 4 | EC | concurrent treatment in clinical trial | concurrent treatment in clinical trial | concurrent treatment in clinical trial |
| EWING 2008 | 5 | EC | Diagnosis: any other medical condition | Diagnosis: any other medical condition | Diagnosis: any other medical condition |
