# Supplementary material for: Leveraging the EHR4CR platform to support patient inclusion in academic studies: challenges and lessons learned
Source: BMC Med Res Methodol. 2017 Feb 28;17:36. doi: 10.1186/s12874-017-0299-3 (PMC5329914; doi:10.1186/s12874-017-0299-3)
Supplement: Additional file 3: — HEGP-CDW Data Completeness for each Medical Concept Individualized During the Normalization Process (DOCX 28 kb) [file 12874_2017_299_MOESM3_ESM.docx]

Additional File 3: HEGP-CDW Data Completeness for each Medical Concept Individualized During the Normalization Process

| N | Individualized medical concepts | Structured/ Unstructured Data | Patient (Completeness^a^) |
| --- | --- | --- | --- |
| 1 | Age | Structured | 84,977 (100%) |
| 2 | Cancer diagnosis | Structured | 5,739 (6.8%) |
| 3 | Active cancer | Unstructured | _ |
| 4 | Histological | Structured | 9,561 (11.3%) |
| 5 | Cytological | Structured | 9,561 (11.3%) |
| 6 | Tumor markers | Structured | 2,337 (2.8%) |
| 7 | Deep vein thrombosis of lower extremity | Structured | 291 (0.3%) |
| 8 | CT angiography of lower limbs | Structured | 158 (0.2%) |
| 9 | Lower limbs phlebography | Structured | 161 (0.2%) |
| 10 | lower limbs venous ultrasonography doppler | Structured | 609 (0.7%) |
| 11 | Compressibility | Unstructured | _ |
| 12 | Venous segment | Unstructured | _ |
| 13 | Veinous lacuna | Unstructured | _ |
| 14 | Vena cava thrombosis | Structured | 36 (0%) |
| 15 | Iliac vein thrombosis | Structured | 25 (0%) |
| 16 | Abdominal CT scan with contrast | Structured | 826 (1%) |
| 17 | Ilio-cavography | Structured | 48 (0.1%) |
| 18 | Pulmonary embolism | Structured | 615 (0.7%) |
| 19 | CT scan of the chest with contrast | Structured | 1,164 (1.4%) |
| 20 | lung Radionuclide imaging | Structured | 303 (0.4%) |
| 21 | Cardiac ultrasound | Structured | 8,374 (9.9%) |
| 22 | Pulmonary artery | Unstructured | _ |
| 23 | Segmental artery | Unstructured | _ |
| 24 | Subsegmental artery | Unstructured | _ |
| 25 | Multiple lacuna | Unstructured | _ |
| 26 | High probability | Unstructured | _ |
| 27 | Dyspnea | Unstructured | _ |
| 28 | Chest pain | Unstructured | _ |
| 29 | Tachycardia | Unstructured | _ |
| 30 | Malaise | Unstructured | _ |
| 31 | Hemoptysis | Unstructured | _ |
| 32 | Pulmonary heart disease | Unstructured | _ |
| 33 | Low-molecular-weight heparin | Structured | 9,156 (10.8%) |
| 34 | Thrombin inhibitor | Structured | 0 |
| 35 | Direct factor Xa inhibitors | Structured | 0 |
| 36 | Creatinine clearance rate | Structured | 40,907 (48.1%) |
| 37 | Pregnancy | Structured | 5 (0%) |
| 38 | Contraception | Structured | 73 (0.1%) |
| 39 | Breast feeding | Structured | 0 (0%) |
| 40 | Weight | Structured | 29,792 (35.1%) |
| 41 | Social insurance, welfare support | Structured | 0 (0%) |
| 42 | Radiation therapy | Structured | 1,321 (1.6%) |
| 43 | Antineoplastic agents | Structured | 1,728 (2.0%) |
| 44 | Heparin allergy | Structured | 0 (0%) |
| 45 | Heparin-induced thrombocytopenia | Structured | 85 (0.1%) |
| 46 | Hemorrhage | Structured | 32 (0%) |
| 47 | Type 2 diabetes mellitus | Structured | 2,355 (2.8%) |
| 48 | Menopause | Structured | 0 (0%) |
| 49 | Oral contraception | Structured | 225 (0.3%) |
| 50 | Intrauterine device | Structured | 0 (0%) |
| 51 | Diaphragm | Unstructured | _ |
| 52 | Spermicide | Structured | 0 (0%) |
| 53 | Female infertility | Structured | 28 (0%) |
| 54 | Hysterectomy | Structured | 0 (0%) |
| 55 | Bilateral tubal ligation | Unstructured | _ |
| 56 | Diabetic Nephropathy | Structured | 323 |
| 57 | Diabetic retinopathy | Structured | 257 (0.3%) |
| 58 | Hematuria | Structured | 78 (0.1%) |
| 59 | Protein/Creatinine Ratio Urine | Missing | _ |
| 60 | Angiotensin-converting-enzyme inhibitors | Structured | 4,003 (4.7%) |
| 61 | Angiotensin II receptor antagonists | Structured | 2,743 (3.2%) |
| 62 | Diuretics | Structured | 5,583 (6.6%) |
| 63 | Renal artery angioplasty | Structured | 0 (0%) |
| 64 | Renal artery stenting | Structured | 59 (0.1%) |
| 65 | Renal artery ultrasonography, doppler | Structured | 654 (0.8% |
| 66 | Renal artery spiral computed tomography angiography | Structured | 3,582 (4.2%) |
| 67 | Renal artery stenosis | Structured | 145 (0.2%) |
| 68 | Systolic pressure | Structured | 1,713 (2.0%) |
| 69 | Diastolic pressure | Structured | 1,704 (2.0%) |
| 70 | Iodine allergy | Structured | 19 (0%) |
| 71 | Nephrogenic fibrosis | Unstructured | _ |
| 72 | Magnetic resonance imaging contrast media | Structured |  |
| 73 | Pacemaker | Structured | 373 (0.4%) |
| 74 | Metalic object | Unstructured | _ |
| 75 | Claustrophobia | Structured | 3 (0%) |
| 76 | Defibrillators, implantable | Structured | 189 (0.2%) |
| 77 | Cholesterol embolism syndrome | Structured | 0 (0%) |
| 78 | Solitary kidney | Structured | 5 (0%) |
| 79 | Renal atrophy | Structured | 3 (0%) |
| 80 | Multiple renal arteries | Structured | 0 (0%) |
| 81 | Aortic prosthesis | Structured | 247 (0.3%) |
| 82 | Severe aortoiliac disease | Structured | 788 (0.9%) |

^a^ The number of distinct patients for which the information was present in the clinical data warehouse of George Pompidou European Hospital (HEGP) in 2013 divided by the total number of distinct patients hospitalized at HEGP during the same time period
